# Cycle Stability and Hydration Behavior of Magnesium Oxide and Its Dependence on the Precursor-Related Particle Morphology

**DOI:** 10.3390/nano8100795

**Published:** 2018-10-07

**Authors:** Georg Gravogl, Christian Knoll, Jan M. Welch, Werner Artner, Norbert Freiberger, Roland Nilica, Elisabeth Eitenberger, Gernot Friedbacher, Michael Harasek, Andreas Werner, Klaudia Hradil, Herwig Peterlik, Peter Weinberger, Danny Müller, Ronald Miletich

**Affiliations:** 1 Department of Mineralogy and Crystallography, University of Vienna, Althanstraße 14, 1090 Vienna, Austria; georg.gravogl@tuwien.ac.at (G.G.); ronald.miletich-pawliczek@univie.ac.at (R.M.); 2 Institute of Applied Synthetic Chemistry, TU Wien, Getreidemarkt 9, 1060 Vienna, Austria; christian.knoll@tuwien.ac.at (C.K.); peter.e163.weinberger@tuwien.ac.at (P.W.); 3 Institute of Chemical, Environmental & Biological Engineering, TU Wien, Getreidemarkt 9, 1060 Vienna, Austria; Michael.harasek@tuwien.ac.at; 4 Atominstitut, TU Wien, Stadionallee 2, 1020 Vienna, Austria; jan.welch@tuwien.ac.at; 5 X-ray Center, TU Wien, Getreidemarkt 9, 1060 Vienna, Austria; werner.artner@tuwien.ac.at (W.A.); Klaudia.hradil@tuwien.ac.at (K.H.); 6 RHI-AG, Magnesitstraße 2, 8700 Leoben, Austria; Norbert.freiberger@rhimagnesita.com (N.F.); Roland.nilica@rhimagnesita.com (R.N.); 7 Institute of Chemical Technologies and Analytics, TU Wien, Getreidemarkt 9, 1060 Vienna, Austria; Elisabeth.eitenberger@tuwien.ac.at (E.E); Gernot.friedbacher@tuwien.ac.at (G.F.); 8 Institute for Energy Systems and Thermodynamics, TU Wien, Getreidemarkt 9, 1060 Vienna, Austria; Andreas.werner@tuwien.ac.at; 9 Faculty of Physics, University of Vienna, Boltzmanngasse 5, 1090 Vienna, Austria; Herwig.peterlik@univie.ac.at

**Keywords:** particle morphology, magnesium hydroxide, magnesium carbonate, magnesium oxalate, magnesium oxide, cycle stability, in-situ powder X-ray diffraction (PXRD), hydration reactivity, thermochemical energy storage, thermochemistry

## Abstract

Thermochemical energy storage is considered as an auspicious method for the recycling of medium-temperature waste heat. The reaction couple Mg(OH)_2_–MgO is intensely investigated for this purpose, suffering so far from limited cycle stability. To overcome this issue, Mg(OH)_2_, MgCO_3_, and MgC_2_O_4_·2H_2_O were compared as precursor materials for MgO production. Depending on the precursor, the particle morphology of the resulting MgO changes, resulting in different hydration behavior and cycle stability. Agglomeration of the material during cyclization was identified as main reason for the decreased reactivity. Immersion of the spent material in liquid H_2_O decomposes the agglomerates restoring the initial reactivity of the material, thus serving as a regeneration step.

## 1. Introduction

Energy management is a major challenge for our society, requiring equal measures of political and scientific involvement [1]. Energy supply, sustainable, environmentally benign energy production, and efficient utilization are key issues in managing global energy use [2]. Energy management may, in many cases, be better expressed as ‘heat management’, as heat is the most ubiquitous form of energy. In nearly all types of electrical power plants, as well as in most industrial processes, heat is used as the driving force and operating medium. Within this context, the utilization of waste heat, accounting for two-thirds of overall global energy production, is an extensively investigated field [3]. The use of waste heat flows includes several aspects, one of them being temporal decoupling of waste heat availability and demand, as the two are not necessarily correlated. The necessary storage may be realized using materials for sensible, latent, or thermochemical storage of energy (heat) [4,5,6,7,8,9]. All three energy storage concepts offer advantages in specific areas of application [6,9,10].

Thermochemical energy storage (TCES) features long-term storage, a wide range of compatible temperatures, applicability as a heat pump system, and finally, high energy storage densities [10,11,12,13]. Based on these aspects, medium-temperature waste heat (up to 450 °C and extensively available from industrial processes) is perfectly suitable for TCES systems. An attractive TCES material for medium-temperature applications is the system Mg(OH)_2_–MgO with a storage temperature around 350 °C [14]. Both Mg(OH)_2_ and MgO are industrial base materials and are, therefore, available in large quantities at low prices.

Mg(OH)_2_–MgO as a TCES material is well known for this purpose, with many aspects related to its application in energy storage already investigated in literature. Kinetic investigations of dehydration and rehydration [15,16], mechanistic aspects of the conversion [17,18], modification of the material by additions of lithium salts [16,19,20], by coating or use of composite material [21,22], by dotation [23], and finally also applicability in form of a chemical heat pump [24] were reported. Nonetheless, two key issues preventing industrial application remain unaddressed: First, rehydration reactivity (completeness), and second, the cycle stability. Whereas for the limited cyclability observed thus far, no satisfying solution has been found, the rehydration reactivity is addressed by the addition of lithium salts [16,19,20], which are quite expensive. On a molecular level, reactivity could also be tuned by dotation of Mg(OH)_2_ with Ca^2+^-ions [23].

**Scheme 1:**
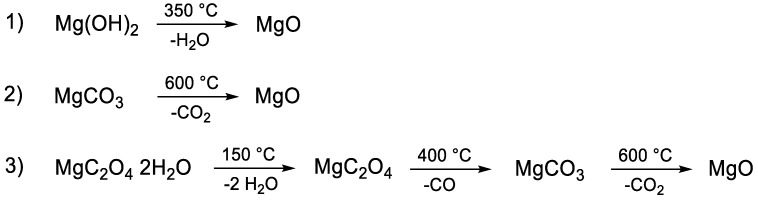
Thermal decomposition of various MgO precursors: (1) Mg(OH)_2_, (2) MgCO_3_, (3) MgC_2_O_4_·2H_2_O.

On an industrial scale MgO is produced via calcination of Mg(OH)_2_ or MgCO_3_ [25]. Both precursors are found in natural deposits, but whereas MgCO_3_ is an industrially mined raw material, Mg(OH)_2_ is produced from serpentinite or processing seawater [26]. However, aerobic calcination of any other Mg compound may result in formation of MgO by stepwise decomposition. In Scheme 1, this is shown at the example of the mentioned industrial precursor, as well as for magnesium oxalate dihydrate.

All so far performed investigations on the rehydration of MgO for thermochemical energy storage purposes have largely neglected the origin of the MgO. As Mg(OH)_2_, MgCO_3_, MgC_2_O_4_·2H_2_O, and MgO crystallize in crystallographically and stereochemically different systems (Table 1), and feature notably different particle morphologies, MgO samples originating from different precursors can not necessarily be expected to have the same properties with respect to rehydration and cycle stability. This assumption is supported by previous kinetic studies on the H_2_O-dissociation on MgO. Compared to the isotypic CaO [27], the lower hydration reactivity of MgO [28] is mainly caused by the kinetic barrier of the water dissociation on the surface [29]. The disfavored H_2_O-dissociation as first step in formation of Mg(OH)_2_ occurs mainly at surface defects, edges, step edges, or corner sites, exhibiting a lower dissociation energy barrier [30]. This suggests that by variation of the particle morphology and origin of the MgO, the rehydration behavior should be affected. While all precursors result in compositionally indistinguishable MgO sample stoichiometries, the particle size and morphology, crystallographic orientation, and thus the orientation of the reactive surfaces of the material are not necessarily the same. To verify this hypothesis, MgO obtained by calcination of Mg(OH)_2_, MgCO_3_, and MgC_2_O_4_·2H_2_O was investigated regarding hydration reactivity and cycle stability.

## 2. Materials and Methods

### 2.1. Material

Mg(OH)_2_ powder (particle size ≤5 µm) and MgCO_3_ (particle size ≤200 µm) were supplied by RHI-AG (X-ray fluorescence analysis (Bruker AXS GmbH, 76187 Karlsruhe, Germany)) of the materials revealed no significant impurities). MgC_2_O_4_·2H_2_O (98.5% purity) was purchased from abcr (GmBH, 76187 Karlsruhe, Germany) and the particle fraction ≤200 µm was used as supplied. The materials were calcined in an electric furnace under air and a static atmosphere for 4 h at variable temperatures (Mg(OH)_2_: at 375 °C; MgCO_3_: at 550 °C, 600 °C, 650 °C; MgC_2_O_4_·2H_2_O: at 650 °C). For subsequent rehydration, the in-situ calcined material from the (powder X-ray diffraction) P-XRD measurement was kept for 24 h in liquid water under ambient pressure-temperature conditions.

### 2.2. BET Surface

The specific surface of the samples was determined by nitrogen sorption measurements, which were performed on an ASAP 2020 (Micromeritics) instrument. The samples (amounting between 100–200 mg) were degassed under vacuum at 80 °C overnight prior to measurement. The surface area was calculated according to Brunauer, Emmett, and Teller (BET, Micromeritics Instrument Corp., Norcross, GA, USA) and t-plot methods [35].

### 2.3. Powder X-ray Diffraction with In-Situ Hydration (P-XRD)

Hydration of calcined samples was performed in an Anton Paar XRK 900 (Bruker AXS GmbH, 76187 Karlsruhe, Germany) sample chamber, connected to an evaporation coil kept at 300 °C (see Figure S1a). Using an HPLC-pump, water was evaporated at rates from 1 g H_2_O min^−1^ up to 3 g min^−1^ and the resulting steam was passed through the sample (1 mm thickness) with 0.2 L min^−1^ nitrogen as carrier gas. The sample is mounted on a hollow ceramic powder sample holder, allowing for complete perfusion of the sample with the water vapour (see Figure S1b). As the sample is completely penetrated by the X-rays, the obtained diffractograms represent an average across the total sample with respect to the quantitative phase proportions. The diffractograms were evaluated using the PANalytical program suite HighScorePlus v3.0d. A background correction and a K_α2_ strip were performed. Phase assignment is based on the ICDD-PDF4+ database (International Diffraction Data-Powder Diffraction File), the exact phase composition, shown in the conversion plots, was obtained via Rietveld-refinement [36] in the program suite HighScorePlus v3.0d. All quantifications based on P-XRD are accurate within of ±2%. The rehydration rates were calculated based on the phase composition derived from the diffractograms, normalizing the percentages of Mg(OH)_2_ and MgO to a total of 100%.

### 2.4. Scanning Electron Microscopy (SEM)

SEM (Thermo Fisher Scientific, 168 Third Avenue, Waltham, MA 02451, USA)) images were recorded on gold coated samples with a Quanta 200 SEM instrument from FEI under low-vacuum at a water vapour pressure of 80 Pa to prevent electrostatic charging.

### 2.5. Small-Angle X-ray Scattering (SAXS)

The samples were prepared either as powder between two pieces of tape or in a sealed capillary. Patterns were recorded using a microsource with X-rays from a copper target (Incoatec High Brilliance, wavelength 0.1542 nm, CuK_α_), a point focus (Nanostar from Bruker AXS) and a 2D detector (VÅNTEC 2000). The X-ray patterns were radially averaged and background corrected to obtain scattering intensities in dependence on the scattering vector *q* = (4π/*λ*) sinθ, with 2θ being the scattering angle.

The fit function from *Beaucage* [37] to describe scattering intensities of complex systems with a broad size distribution consists of a power law and Guinier’s exponential form,

(1) $$I\left(q\right)\propto Gexp\left(\frac{-q^{2}R_{g}^{2}}{3}\right)+B\;{\left[\frac{\left(erf(qR_{g}/\sqrt{6}\right){)}^{3}}{q}\right]}^{d_{f}}$$

where *G* and *B* are the numerical prefactors, *d_f_* is the fractal dimension, *R_g_* is the radius of gyration and *erf*(*x*) is the error function. To describe the particle interference and thus the tendency of particles to agglomerate, additionally a structure factor from a hard sphere model was used [38,39],

(2) $$I\left(q\right)\propto \left(G\exp\left(\frac{-q^{2}R_{g}^{2}}{3}\right)+B\;{\left[\frac{\left(erf(qR_{g}/\sqrt{6}\right){)}^{3}}{q}\right]}^{d_{f}}\right)\;S\left(q\right)$$

with

(3) $$S\left(q\right)=1/\left(1+24\eta \;G_{int}\left(2qR_{HS}\right)/\left(2qR_{HS}\right)\right)$$

and *R_HS_* being the hard sphere radius describing a typical distance of objects, *η* the hard sphere volume factor for characterizing the amount of agglomeration, and *G_int_* a function derived in Kinning et al. [38].

## 3. Discussion and Results

**Figure 1:**
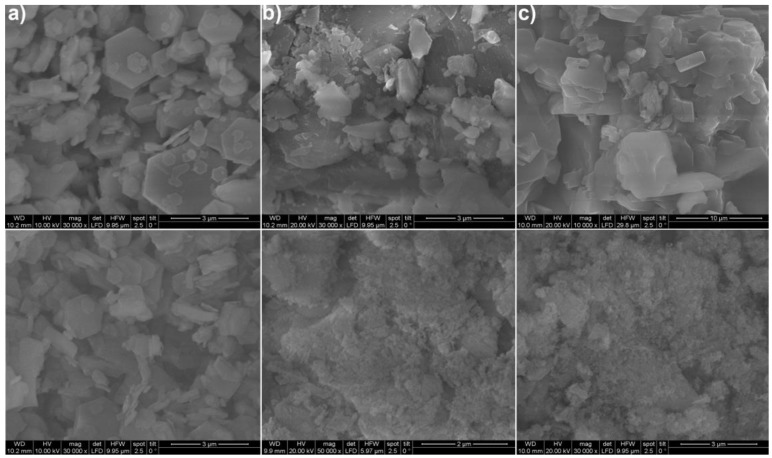
SEM pictures of (**a**) Mg(OH)_2_; (**b**) MgCO_3_; (**c**) MgC_2_O_4_·2H_2_O before calcination (first row) and after calcination (second row).

To combine the apparent particle morphology with the crystallographic features of the lattice as given in Table 1, SEM-images of the original and calcined materials are compared in Figure 1. The first row corresponds to SEM-images of the various MgO precursors; in the second row the resulting MgO samples, obtained after thermal decomposition, are shown. Whereas Mg(OH)_2_ particles feature euhedral idiomorphic shapes with characteristic faces following hexagonal symmetry (Figure 1a), both MgCO_3_ (Figure 1b) and MgC_2_O_4_·2H_2_O (Figure 1c) reveal subidiomorphic irregular particle shapes occasionally showing typical rhombohedral (Figure 1b) or foliated (Figure 1c) cleavage faces, which in the case of MgC_2_O_4_·2H_2_O correspond to its layer structure.

The particle morphology of the materials changes during calcination (Figure 1, second row), leading to three differently textured MgO samples. Whereas for using Mg(OH)_2_ as the precursor material (Figure 1a), calcination results in an apparently unchanged particle morphologies, the MgO crystallites obtained from both MgCO_3_ (Figure 1b) and MgC_2_O_4_·2H_2_O (Figure 1c) precursors are characterized by a clear surface fragmentation, which can be attributed to larger degree of structural reconstruction on the release of volatile components. In contrast, the H_2_O release from Mg(OH)_2_ to MgO follows a simple change from hcp to ccp arrangement of the octahedral subunits and hence preserves the particles in its shape to a large extent.

On the nanoscale, small-angle X-ray scattering (SAXS) reveals a transformation of the material from a dense solid to a highly porous material on calcination (see Figure S2). The nanostructure of MgO was modelled by a unified Guinier/power law [37], resulting in a radius of gyration for the size of the particles and an agglomeration with a structure factor from a hard sphere model, describing the agglomeration of particles with a typical distance 2*R_HS_* and the packing density with a hard sphere volume ratio *η* [38,39]. The detailed fit parameters are found in the supporting information (Table S1). In general, the gyration radius of MgO particles calcined from MgCO_3_ and MgC_2_O_4_·2H_2_O is about 6.6 and 5.1 nm, respectively, in comparison to about 2 nm if calcined from Mg(OH)_2_. In contrast, the values of *η* = 0.18 and a fractal dimension of *d_f_* = 2.8 indicate, that MgO from Mg(OH)_2_ consists of small, agglomerated particles with a wide size distribution, whereas MgO from other precursors is built up of larger, denser nanoparticles (*η* close to zero, *d_f_* = 4).

**Scheme 2:**
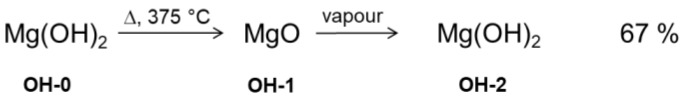
Conditions for calcination and rehydration of MgO obtained from Mg(OH)_2_.

In order to allow a better comparability between the different precursor materials investigated within this study, MgO obtained by calcination from Mg(OH)_2_ was used as reference material [28]. In Scheme 2, schematic representation of the calcination and rehydration conditions applied for its preparation is shown.

**Figure 2:**
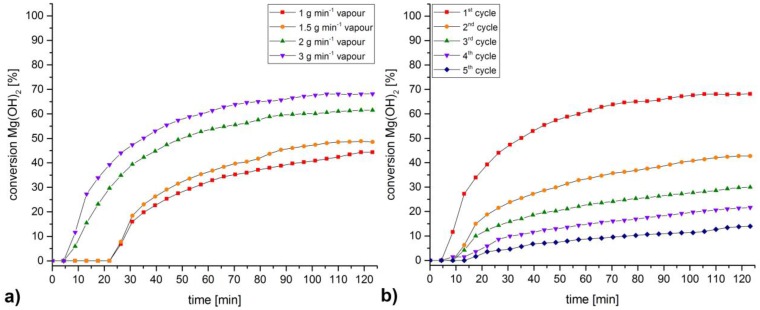
(**a**) Rehydration of Mg(OH)_2_-originating MgO with various water vapour flow rates; (**b**) Cycle stability of Mg(OH)_2_-originating MgO.

To correlate the particle morphologies with rehydration reactivity and cycle stability, rehydration experiments using the different MgO samples were monitored by in situ powder X-ray diffraction (P-XRD). This allows for a direct observation and quantification of the reaction progress. As in previous experiments, the rehydration reactivity of the MgO produced from Mg(OH)_2_ was found quite limited [28]. To eventually increase the reaction rate, an even larger excess of water vapour was introduced into the reaction chamber. Increasing the vapour flow from 1 g min^−1^ to 3 g min^−1^ enhanced the rehydration conversion of MgO from 44% to 67% (Figure 2a). To assess the cycle stability for the increased vapour flow, five consecutive rehydration–calcination cycles were performed (Figure 2b). Similar to previous experiments with lower vapour flows the rehydration yield decreased over 5 cycles to a final Mg(OH)_2_ conversion of only 14%. Even after the first cycle the rehydration conversion was depleted to 43%.

For MgO produced by calcination of Mg(OH)_2_ a strong correlation between reactivity, accessible surface area and calcination temperature has been established [28]. Higher calcination temperatures promote sintering of the particles, leading to a decreased porosity, increased MgO crystal size, and decreased rehydration yield.

**Scheme 3:**
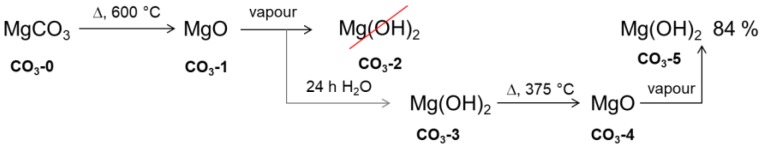
Conditions for calcination and rehydration of MgO obtained from MgCO_3_.

To assess the possibility of a similar effect for MgO originating from MgCO_3_, initial studies of the correlation between calcination temperature, BET surface and rehydration reactivity were made. For this purpose, samples of MgCO_3_ were calcined at 550 °C, 600 °C, and 650 °C for 3, 6, 9, and 12 h. The MgO formed from MgCO_3_ calcined for 6 h at 600 °C had the highest surface area (Figure S3). Nevertheless, attempted rehydration of all samples by water vapour in the P-XRD failed, showing no Mg(OH)_2_ formation within 120 minutes. In Scheme 3, representation of the conditions applied for calcination and rehydration of MgCO_3_-derived MgO is given.

**Figure 3:**
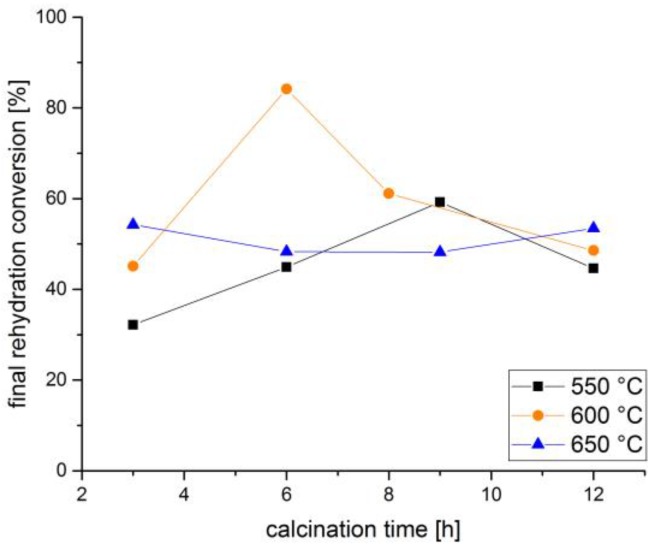
Final conversion for rehydration of the various MgCO_3_-originating MgO samples **CO_3_-4** in the P-XRD.

Based on the assumption, that a different chemical history of the MgO would have an impact on the reactivity during rehydration, a varied rehydration rate would have been expected. Observing no conversion to Mg(OH)_2_ under the applied conditions was, however, quite unexpected. On prolonged exposure to water vapour over 24 h for MgO **CO_3_-1** (see Scheme 3) a very sluggish formation of Mg(OH)_2_ below 10% was observed. To ascertain whether rehydration of this material could be driven by longer exposure to a vast excess of reactant, the samples were stored in liquid water. After 24 h reaction time in water at room-temperature, according to P-XRD measurements the material had been completely transformed to Mg(OH)_2_ (**CO_3_-3**) To repeat the in situ rehydration study for this material, the calcination step was repeated at 375 °C using the conditions developed for Mg(OH)_2_ [28]. After a new calcination step the BET surface of the various samples **CO_3_-4** was found to be slightly higher than for **CO_3_-1**, the MgO originating directly from MgCO_3_ (Figure S4). In contrast to the first attempt, now for those materials the rehydration experiments in the P-XRD were repeated successfully for all materials (for detailed rehydration rates see Figure S5). Ranked according to their final conversion to Mg(OH)_2_, the most reactive material within this series was obtained by calcination of MgCO_3_ at 600 °C for 6 h (Figure 3) and subsequent rehydration in liquid water. A final conversion to 84% Mg(OH)_2_ was not only by far the highest yield for the MgCO_3_-originating series, but also notably more than for MgO originating from Mg(OH)_2_ (67% final conversion).

**Figure 4:**
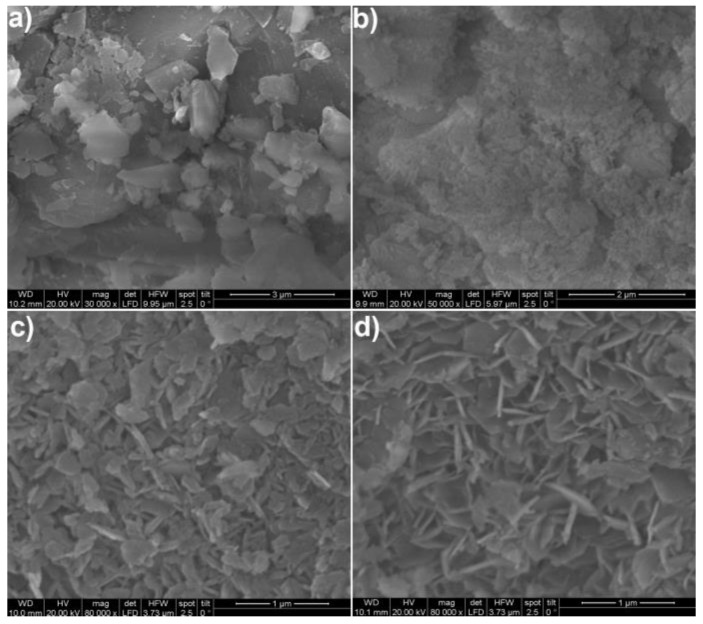
SEM images of (**a**) MgCO_3_, (**b**) calcined MgCO_3_ (**CO_3_-1**), (**c**) calcined MgCO_3_, rehydrated for 24 h in liquid water (**CO_3_-3**), (**d**) material from image c after calcination (**CO_3_-4**).

SEM images demonstrate, that the particle morphology of MgCO_3_ (Figure 4a) is retained after calcination, although the formerly distinct edges and surfaces are now covered by smaller scales (Figure 4b). During the hydration of the calcined material in liquid water the large particles disintegrate into smaller platelets (Figure 4c), although lacking the characteristic hexagonal morphology as characteristic for euhedrally grown Mg(OH)_2_ (see Figure 1). A subsequent calcination of material rehydrated in liquid water retains the afore mentioned platelet morphology (Figure 4d).

The changing particle shape observed in the SEM images is attributed to the volume work going along with the Mg(OH)_2_ formation. To enable this rehydration-related rearrangement of the material, water in its liquid form seems crucial as an agent triggering the rehydration process. SAXS intensities (Figure S6) show that on (repeated) rehydration of the MgCO_3_-derived MgO-samples the particle morphology is widely unchanged. From the larger scattering intensity a highly porous nanostructure, retained during rehydration, may be extrapolated. At the same time, a general decrease in particle size was also observed, being in good agreement with the SEM images (Figure 4).

**Figure 5:**
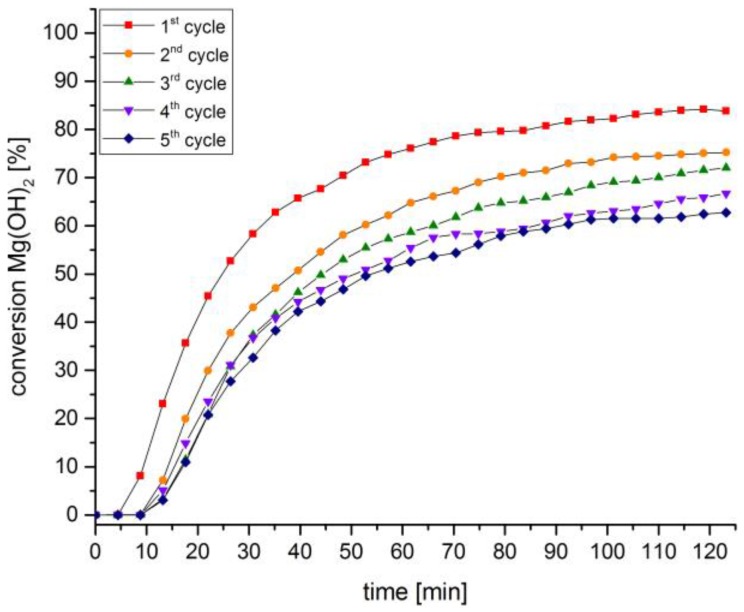
Cycle stability of MgCO_3_-originating MgO **CO_3_-4**.

The carbonate-derived MgO **CO_3_-4** was also investigated in terms of cycle stability (Figure 5). Similar to the material **OH-1** originating from Mg(OH)_2_, also in the case of **CO_3_-4** a decrease in rehydration reactivity was detected, although to a lesser extent than observed for **OH-1**. Over five cycles the rehydration conversion drops to 57% (84% in the 1st, 75% in the 2nd cycle).

**Scheme 4:**
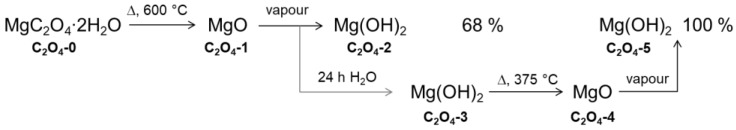
Conditions for calcination and rehydration of MgO obtained from MgC_2_O_4_·2H_2_O.

As a third precursor for preparation of reactive MgO, MgC_2_O_4_·2H_2_O was investigated (see Scheme 4).

**Figure 6:**
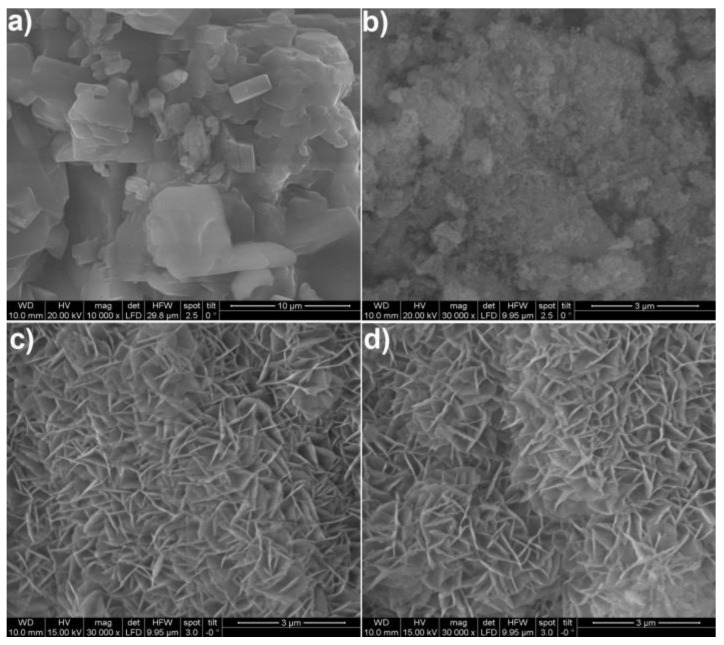
SEM images of (**a**) MgC_2_O_4_·2H_2_O, (**b**) calcined MgC_2_O_4_·2H_2_O (**C_2_O_4_-1**), (**c**) calcined MgC_2_O_4_·2H_2_O, rehydrated for 24 h in liquid water (**C_2_O_4_-3**), (**d**) material from image **c** after calcination (**C_2_O_4_-4**).

Since MgC_2_O_4_·2H_2_O decomposes stepwise via MgCO_3_ (see Scheme 1), only samples calcined in the furnace at 600 °C for 6 h were investigated. A comparison of the SEM images in Figure 6, compares the morphology of the different samples: initial oxalate material **C_2_O_4_-0** (Figure 6a), the calcined material **C_2_O_4_-1** (Figure 6b), MgO after rehydration in liquid water **C_2_O_4_-3** (Figure 6c), and a new calcined material **C_2_O_4_-4** (Figure 6d). Similar to the MgCO_3_-case, calcination of the initial material resulted in partial fragmentation, whereas subsequent treatment with liquid water and re-calcination forced the material to adopt a lamellar-structured particle morphology. In contrast to the MgO originating from MgCO_3_, thinner platelets were formed, those structure is preserved after calcination. Moreover, even the rehydration of the oxalate-based MgO did not yield the typical hexagonally shaped morphologies of euhedral brucite crystallites.

Both SEM and SAXS data show a comparable picture as observed for MgCO_3_. Rehydration of MgO **C_2_O_4_-1** in liquid water results in conservation of the original particle morphology to a wide extent (Figure S7). The porous nanostructure formed is preserved during rehydration. Accordingly, the increased SAXS intensities observed for the rehydrated material seem to arise from the formation of smaller particles (SEM images, Figure 6).

**Figure 7:**
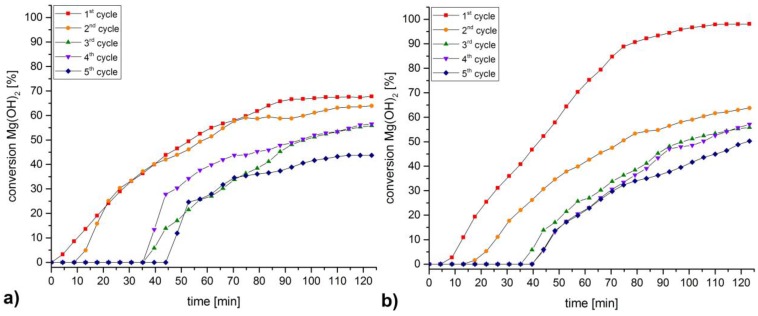
(**a**) Direct rehydration and cycle stability of MgC_2_O_4_-originating MgO **C_2_O_4_-1**; (**b**) Rehydration and cycle stability of MgC_2_O_4_-originating MgO **C_2_O_4_-4**.

To determine the reactivity of the MgC_2_O_4_·2H_2_O-based MgO, both the material obtained by direct calcination of MgC_2_O_4_·2H_2_O (**C_2_O_4_-1**) and that resulting from the rehydration–calcination sequence (**C_2_O_4_-4**) were subject to rehydration in the P-XRD. Unlike the case of MgCO_3_, the directly calcined material **C_2_O_4_-1** was found to be reactive to rehydration, resulting in a final conversion of 68% (Figure 7a).

The most remarkable finding is, that several batches of **C_2_O_4_-4**, the sample previously rehydrated in liquid H_2_O and subsequently calcined, could be fully rehydrated in the 1st cycle, but in the 2nd cycle the rehydration conversion decreased to 64% (Figure 7b). After five cycles both samples gave a comparable final conversion of slightly less than 50% Mg(OH)_2_.

Assessing the technological feasibility of MgC_2_O_4_·2H_2_O as precursor material, on the one hand the material was completely rehydrated to Mg(OH)_2_ within the first cycle after an initial treatment with liquid water. On the other hand, due to a large decrease in conversion rate during the successive cycles, a modest overall performance, and a relatively higher price compared to Mg(OH)_2_ and MgCO_3_, MgC_2_O_4_·2H_2_O is most likely not suitable as a competitive MgO precursor. Therefore, MgC_2_O_4_·2H_2_O was not subjected further studies.

**Figure 8:**
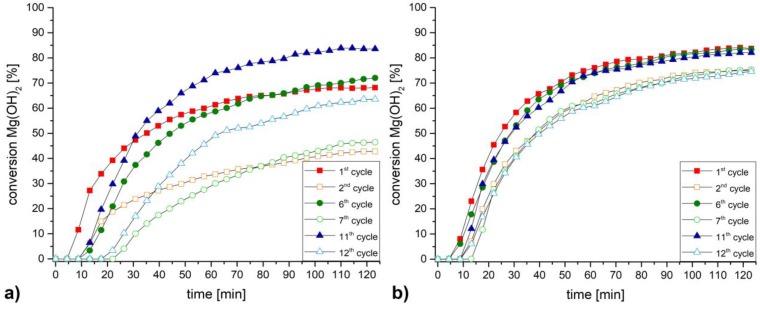
Selected conversion rates from a series of 15 consecutive calcination–hydration cycles, including two regeneration steps in liquid water after the 5th and the 10th cycle. (**a**) Mg(OH)_2_-originating MgO after regeneration; (**b**) MgCO_3_-originating MgO after regeneration.

Based on the conversion-enhancing effect of rehydrating calcined material in liquid water, both a sample of spent MgO originating from Mg(OH)_2_ and one from MgCO_3_ after the 5th rehydration cycle was calcined a further time and then rehydrated for 24 h in liquid water. At that point, P-XRD analysis showed complete transformation to Mg(OH)_2_ for both samples. Both Mg(OH)_2_-samples were now subjected further five rehydration–calcination cycles in the P-XRD, followed by a further regeneration in liquid water and another five rehydration–calcination cycles in the P-XRD. The conversion rates for 15 consecutive cycles, including two regeneration steps after five cycles are shown in Figure 8.

In the case of MgO originating from Mg(OH)_2_, shown in Figure 8a, the conversion rate after the first regeneration (Cycle 6) was slightly enhanced compared to the very 1st cycle. This effect was even more pronounced after the second regeneration (Cycle 10), revealing an even further increased reactivity. Nevertheless, the depletion evidenced in the second cycle was retained even after the second cycles after regeneration, as observed for Cycles 6 and 12. These results could be reproduced on various batches.

In the case of MgO derived from MgCO_3_, a different but even more promising effect was observed: The spent material could be completely regenerated to reproduce the reactivity observed for the first cycle. Even the second cycle after each regeneration process (Cycle 7 and 12) was comparable to the “first” second cycle. Even in this case the effect was reproducible on various batches.

**Figure 9:**
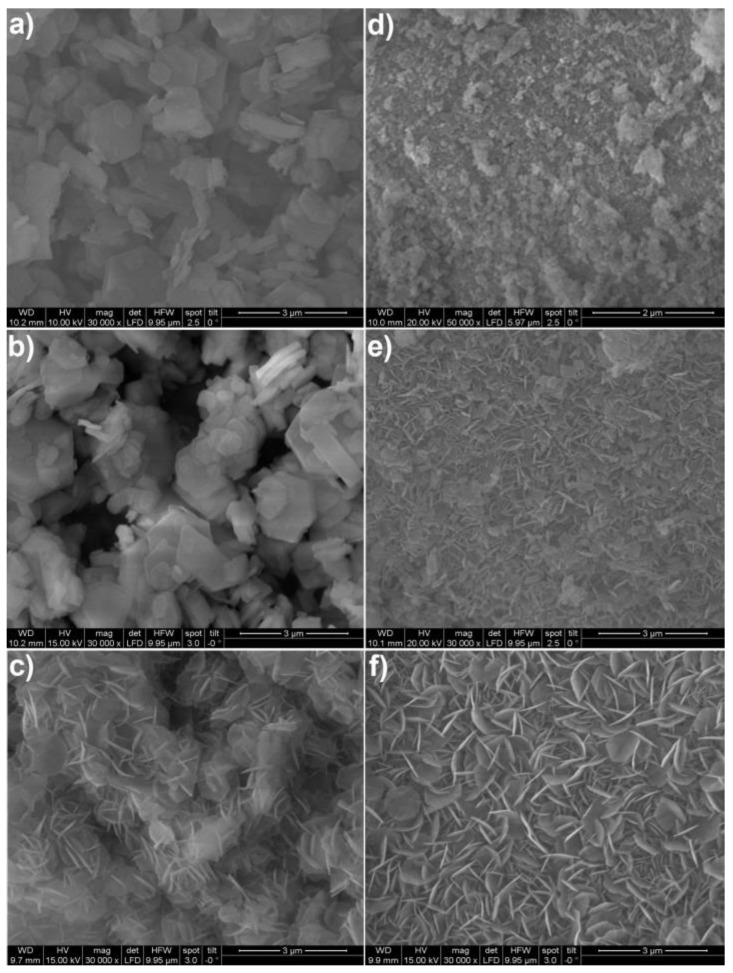
SEM images of various intermediates during calcination/rehydration/regeneration for Mg(OH)_2_- (left) and MgCO_3_-originating MgO (right); (**a**) Mg(OH)_2_-originating MgO; (**b**) Mg(OH)_2_-originating MgO after 5 rehydration–calcination cycles; (**c**) material of image b after regeneration for 24 h in liquid H_2_O; (**d**) MgCO_3_-originating MgO; (**e**) MgCO_3_-originating MgO after five rehydration–calcination cycles; (**f**) material of image **e** after regeneration for 24 h in liquid H_2_O.

To better understand the physical processes during regeneration, SEM images of material during several stages of regeneration and cycling were compared. Despite differences in initial particle morphology (Figure 9a,d), MgO originating from Mg(OH)_2_ or MgCO_3_ shows similar evolution of reactivity during repeated calcination–rehydration cycles. After five consecutive cycles, resulting in aged material of depleted rehydration reactivity (see Figure 2 and Figure 5), the particle morphology of Mg(OH)_2_-derived MgO (Figure 9b) seems nearly unaffected. In contrast, for material originating from MgCO_3_, the larger spherical aggregates are retained (Figure 9e). After regeneration for 24 h in liquid water both materials reveal a lamellar, platelet morphology devoid of the characteristic hexagonal brucite particle shape (Figure 9c,f).

To directly monitor the rehydration/regeneration process, both a sample of MgO originating from Mg(OH)_2_ and from MgCO_3_ were observed during rehydration in liquid water by in situ SAXS (Figures S8 and S9). Initial SAXS curves (black) and final SAXS curves (red) are highlighted to better visualize the data and clearly show the change in the inner structure of the particles. The main difference is the time required for structural recovery, which is about three-fold shorter for Mg(OH)_2_-derived material compared to that from MgCO_3_ (Figure S8), most likely due to the considerably larger particle sizes favored by the latter.

Within various repeated experiments the regeneration process described for spent MgO was found to be reproducible—on one hand for the material of the same origin, on the other for materials of different origins. We suggest that during the regeneration process due to the comparably long reaction time and the vast excess of water, a complete conversion to Mg(OH)_2_ as well as a regeneration of the particle morphology occurs. Both effects complement each other, restoring the original reactivity of the material.

The possibility of a regeneration of spent material is of utmost importance for assessing the economic feasibility of a TCES material and energy storage process, as by prolonging the life-time the materials investment costs are minimized. Additionally, by implementation of a continuous regeneration step into the process, regenerating after each discharging–charging cycle a defined amount of material, permanent high activity of the TCES material circumventing efficiency losses by ageing would be ensured.

## 4. Conclusions

MgO obtained by calcination of Mg(OH)_2_, MgCO_3_, and MgC_2_O_4_·2H_2_O was compared regarding its rehydration reactivity and cycle stability to assess its applicability in thermochemical energy storage. The three different MgO-precursors led to three MgO samples featuring different particle morphologies with identical chemical compositions. Whereas Mg(OH)_2_ and MgC_2_O_4_·2H_2_O resulted in reactive MgO that could be rehydrated by water vapour to Mg(OH)_2_ directly following calcination, material originating from MgCO_3_ resulted in no conversion on contact with water vapour. Only after rehydration in liquid water and subsequent calcination of the thus formed Mg(OH)_2_, 84% of the resulting material could be rehydrated by water vapour. All materials investigated showed decreased rehydration reactivity during consecutive calcination–rehydration cycles, with MgCO_3_-derived MgO showing the smallest decline in reactivity. A regeneration step, consisting of rehydration of the spent material in liquid water over 24 h, restored the initial reactivity allowing for recycling of the material. In the case of Mg(OH)_2_ derived material, the initial reactivity could even be improved by repeated regeneration of the material in liquid water.

The results reported herein confirm, that the reactivity of MgO towards rehydration is strongly correlated to origin and physicochemical history of the material–an aspect so far neglected in the research on TCES materials. The correlation between chemical history and performance of storage materials may stimulate additional to coating, chemical dotation, etc., the consideration of a further, easily tunable parameter for the research on novel TCES systems.

## Figures and Tables

**Table 1:** Comparison of the crystallographic parameters of selected MgO precursors and MgO.

	Mg(OH)_2_ [31]	MgCO_3_ [32]	MgC_2_O_4_·2H_2_O [33]	MgO [34]
Space group	P$\bar{3}$m1 (164)	R$\bar{3}$c (167)	Fddd (70)	Fm$\bar{3}$m (225)
a [Å]	3.1486(1)	4.637(1)	5.3940(11)	4.2113(5)
b [Å]	3.1486(1	4.637(1)	12.691(3)	4.2113(5)
c [Å]	4.7713(1)	15.023(3)	15.399(3)	4.2113(5)
α [°]	90	90	90	90
γ [°]	120	120	90	90
V [Å^3^]	40.96	279.74	1054.14	74.69

## Supplementary Materials

The following are available online at http://www.mdpi.com/2079-4991/8/10/795/s1, Figure S1: Rehydration setup, reaction chamber and sample holder used for the in situ studies. Figure S2: SAXS intensities of starting materials and materials after calcination. Figure S3: BET surfaces of the MgCO_3_-originating MgO samples. Figure S4: BET surfaces of the MgCO_3_-originating MgO samples after rehydration. Figure S5: Rehydration rates of MgCO_3_-originating MgO samples in the P-XRD. Figure S6: SAXS intensities of materials from MgCO_3_ precursor. Figure S7: SAXS intensities of materials from Mg_2_C_2_O_4_·2H_2_O precursor. Figure S8: In situ SAXS intensities during regeneration in liquid water for 24 h. Figure S9: Kinetics of conversion to hydroxide during regeneration in liquid water. Table S1: Fit data for calcined materials.
