# Supplementary material for: Cycle Stability and Hydration Behavior of Magnesium Oxide and Its Dependence on the Precursor-Related Particle Morphology
Source: Nanomaterials (Basel). 2018 Oct 7;8(10):795. doi: 10.3390/nano8100795 (PMC6215189; doi:10.3390/nano8100795)
Supplement: Supplementary file 1 [file nanomaterials-08-00795-s001.pdf]

nanomaterials

MDPI

Supporting information

Cycle Stability and Hydration Behavior of Magnesium Oxide and Its Dependence on The Precursor-Related Particle Morphology

Georg Gravogl [1,2], Christian Knoll [2,3], Jan M. Welch [4], Werner Artner [5], Norbert Freiberger [6], Roland Nilica [6], Elisabeth Eitenberger [7], Gernot Friedbacher [7], Michael Harasek [3], Andreas Werner [8], Klaudia Hradil [5], Herwig Peterlik [9], Peter Weinberger [2], Danny Müller [2,*] and Ronald Miletich[1]

[1] Department of Mineralogy and Crystallography, University of Vienna, Althanstraße 14, 1090 Vienna, Austria; georg.gravogl@tuwien.ac.at (G.G.); ronald.miletich-pawliczek@univie.ac.at (R.M.)
[2] Institute of Applied Synthetic Chemistry, TU Wien, Getreidemarkt 9, 1060 Vienna, Austria; christian.knoll@tuwien.ac.at (C.K.); peter.e163.weinberger@tuwien.ac.at (P.W.)
[3] Institute of Chemical, Environmental & Biological Engineering, TU Wien, Getreidemarkt 9, 1060 Vienna, Austria; Michael.harasek@tuwien.ac.at (M.H.)
[4] Atominstitut, TU Wien, Stadionallee 2, 1020 Vienna, Austria; jan.welch@tuwien.ac.at (J.M.W.);
[5] X-Ray Center, TU Wien, Getreidemarkt 9, 1060 Vienna, Austria; werner.artner@tuwien.ac.at (W.A.); Klaudia.hradil@tuwien.ac.at (K.H.)
[6] RHI-AG, Magnesitstraße 2, 8700 Leoben, Austria; norbert.freiberger@rhimagnesita.com (N.F.); Roland.nilica@rhimagnesita.com (R.N.)
[7] Institute of Chemical Technologies and Analytics, TU Wien, Getreidemarkt 9, 1060 Vienna, Austria; Elisabeth.eitenberger@tuwien.ac.at (E.E); Gernot.friedbacher@tuwien.ac.at (G.F.)
[8] Institute for Energy Systems and Thermodynamics, TU Wien, Getreidemarkt 9, 1060 Vienna, Austria; Andreas.werner@tuwien.ac.at
[9] Faculty of Physics, University of Vienna, Boltzmanngasse 5, 1090 Vienna, Austria; Herwig.peterlik@univie.ac.at (H.P.)
* Correspondence: danny.mueller@tuwien.ac.at; Tel.: +43-1-58801-163740

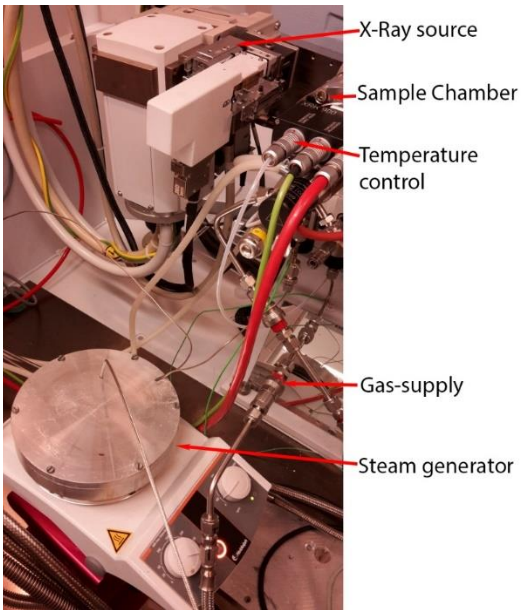

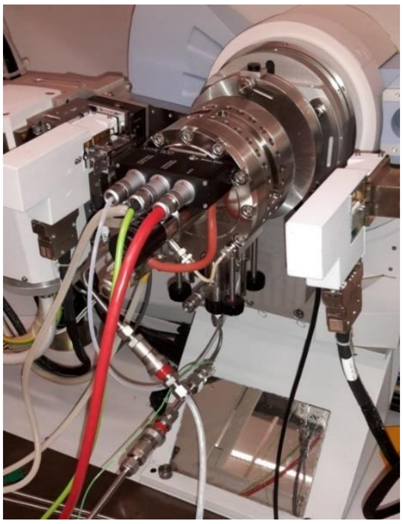

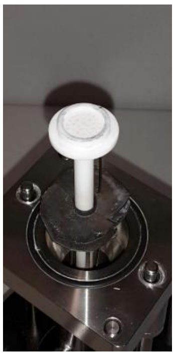

Figure S1a. Rehydration setup in the P-XRD

Figure S1b. Reaction chamber and sample holder used for the in-situ experiments

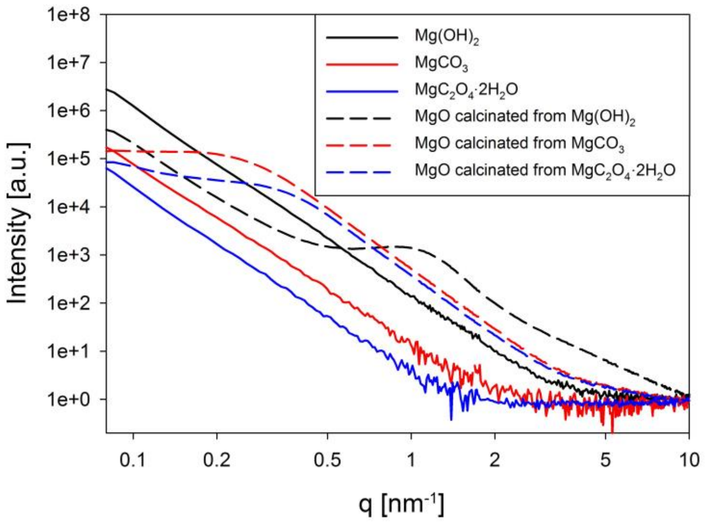

Figure S2. SAXS intensities of starting materials and materials after calcination. The considerably higher scattering intensity after calcination shows the development into a porous material consisting of particles in the nanometer size.

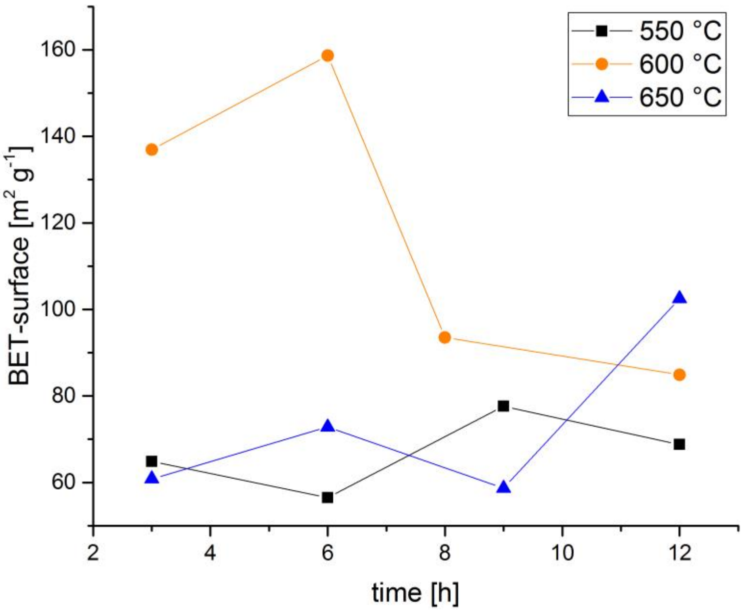

Figure S3. BET-surfaces of the $MgCO_3$-originating MgO-samples.

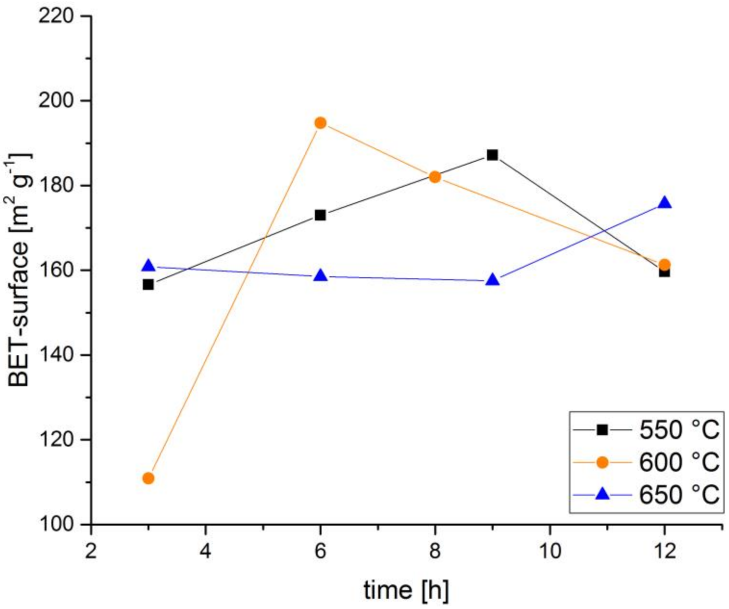

Figure S4. BET-surfaces of the $MgCO_3$-originating MgO-samples after rehydration for 24 h in liquid water and subsequent calcination.

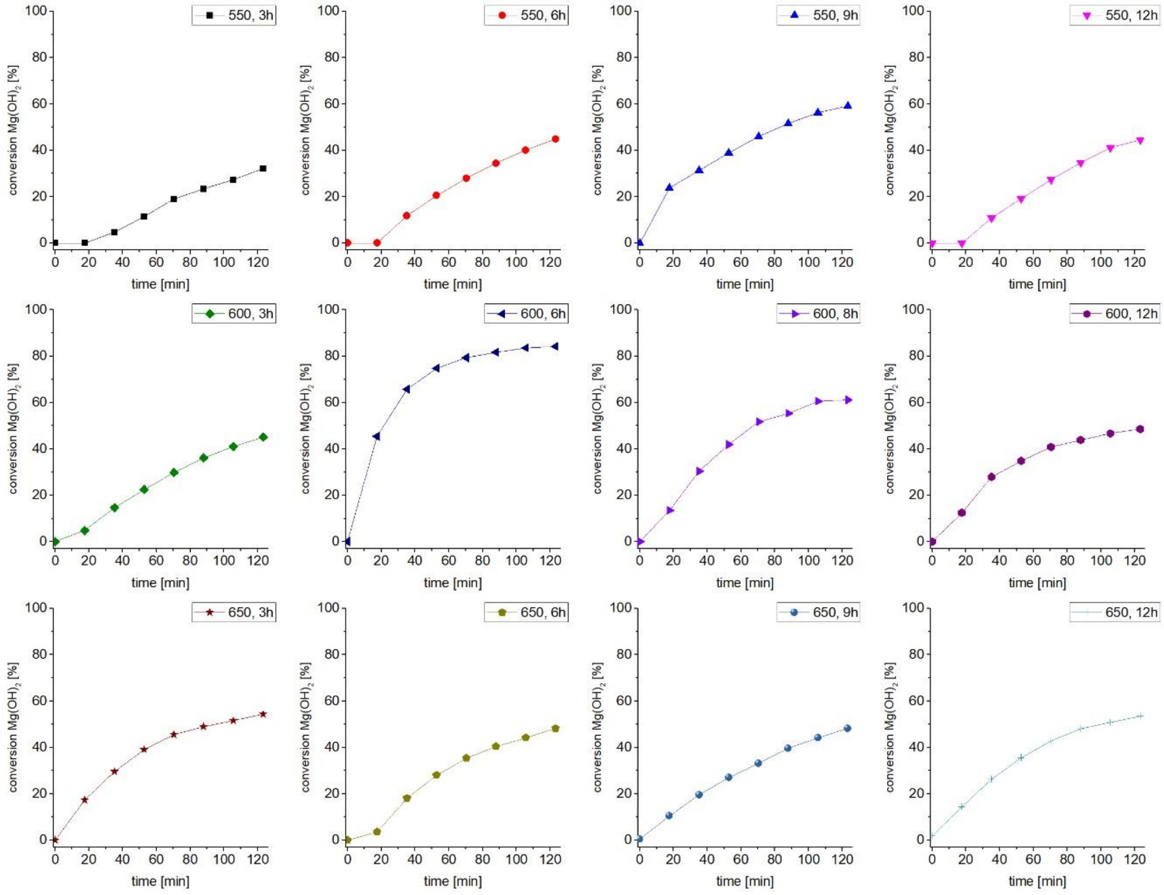

Figure S5. Rehydration rates of $MgCO_3$-originating MgO-samples in the P-XRD after rehydration for 24 h in liquid water and subsequent calcination.

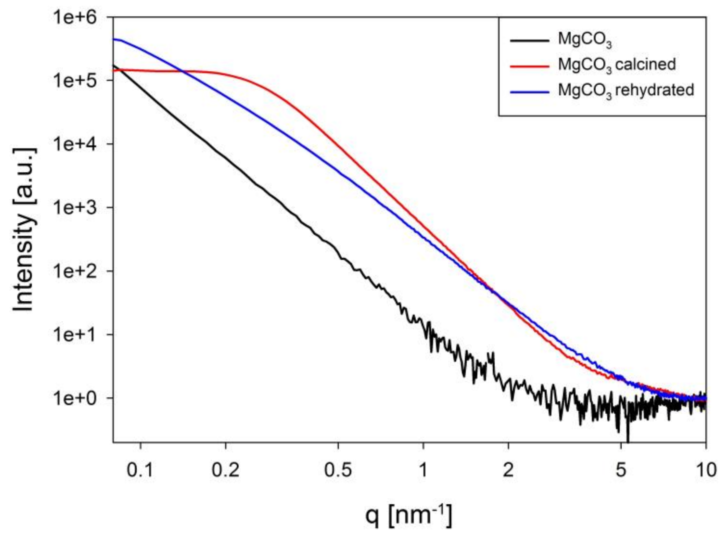

Figure S6. SAXS intensities of materials from $MgCO_3$ precursor. The original structure is recovered to a wide extent after rehydration. The porosity is, however, is much higher after calcination and rehydration, visible by the higher scattering intensity.

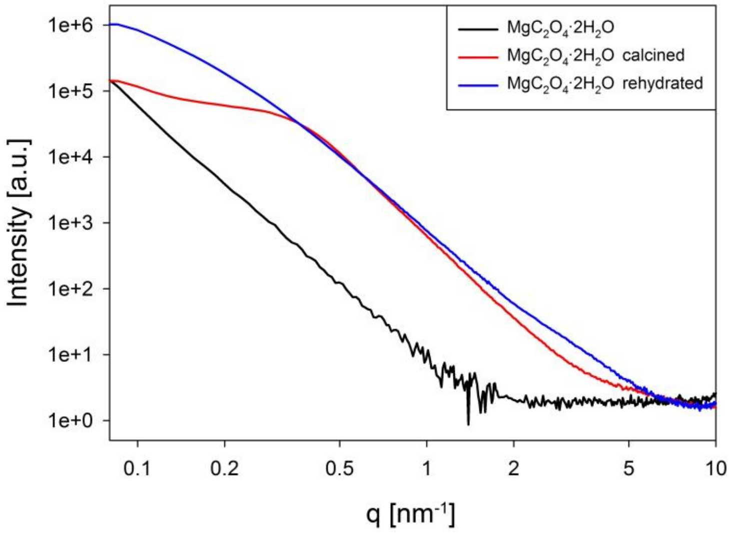

Figure S7. SAXS intensities of materials from $Mg_2C_2O_4{\cdot}2H_2O$ precursor. The original structure is recovered to a wide extent after rehydration. The porosity, however, is much higher after calcination and rehydration, visible by the higher scattering intensity.

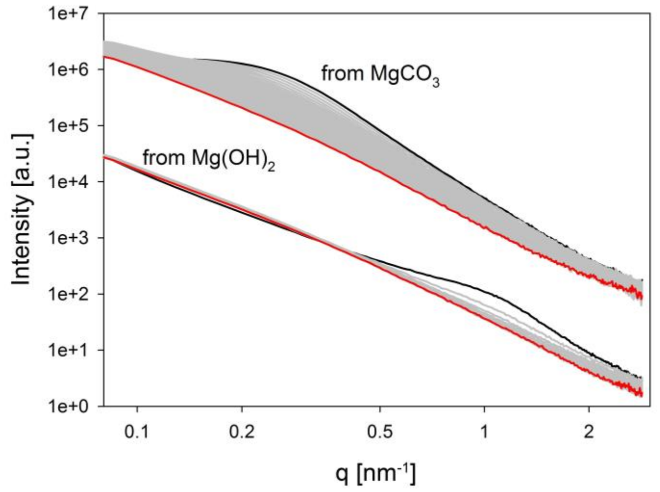

Figure S8. *In-situ* SAXS intensities during regeneration in liquid water for 24 h. SAXS curve at the beginning in black, then grey lines for measurements taken each half an hour and final measurement (red line). The $MgCO_3$ lines vertically shifted for better visibility. A considerable difference is the much faster kinetics for the $Mg(OH)_2$ derived material.

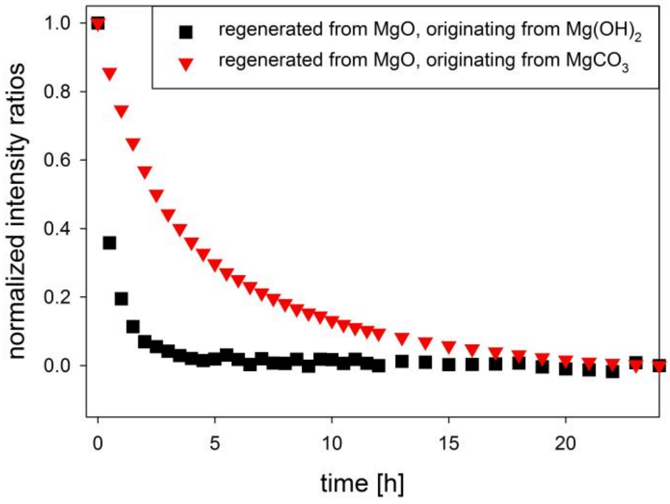

Figure S9. Kinetics of conversion to hydroxide during regeneration in liquid water, evaluated from the decrease of the intensity difference between starting (calcined) and finally transformed hydroxide material from *in-situ* SAXS intensity data in Fig S1d, normalized to the initial difference. Regeneration kinetics is about three times slower for $MgCO_3$ than for $Mg(OH)_2$ originating material.

Table S1. using a model combining the unified scattering function of Beaucage /1/ (resulting in a radius of gyration R_g and a fractal dimension d_f) together with a packing factor from a hard sphere model /2,3/ describing the agglomeration of units (distance R_{HS} and hard sphere volume ratio η).

Material MgO	R_g / nm	d_f	R_{HS} / nm	η
calcined from $(Mg(OH)_2$	1.8	2.8	2.6	0.18
calcined from $MgCO_3$	6.6	4.0	12	0.06
calcined from $Mg_2C_2O_4.2H_2O$	5.1	4.0	7.0	0.04
